# Does Modification of Amalgomer with Propolis Alter Its Physicomechanical Properties? An In Vitro Study

**DOI:** 10.1155/2020/3180879

**Published:** 2020-05-11

**Authors:** Reham M. Abdallah, Amr M. Abdelghany, Neven S. Aref

**Affiliations:** ^1^Dental Biomaterials Department, Faculty of Dentistry, Mansoura University, Mansoura, Egypt; ^2^Dental Biomaterials Department, Faculty of Dentistry, Horus University, New Damietta, Dumyat al Jadida, Egypt; ^3^Spectroscopy Department, Physics Division, National Research Center, Dokki Cairo, Giza, Egypt; ^4^Basic Oral and Medical Sciences Department, College of Dentistry, Qassim University, Buraydah, Saudi Arabia

## Abstract

**Objective:**

To assess if incorporating ethanolic extract of propolis into ceramic-reinforced glass ionomer (Amalgomer CR) might have an influence on its physicomechanical properties.

**Materials and Methods:**

Three groups were assessed; group I: Amalgomer CR (control) and two experimental groups (II and III) of propolis added to the liquid of Amalgomer CR with 25 and 50 v/v %, respectively. Evaluation parameters were color stability, compressive strength, microhardness, and surface roughness. Representative specimens of each group were analyzed by Fourier-transform infrared spectroscopy, energy-dispersive X-ray, X-ray diffraction, and scanning electron microscopy. Analysis of variance (ANOVA) was used to compare the results, followed by a Tukey post hoc test (*p* < 0.05).

**Results:**

Nonsignificant color change for both groups of modified Amalgomer CR. Meanwhile, the two experimental groups exhibited a significant increase in both compressive strength and microhardness. Simultaneously, there was a significant difference in roughness values among groups with the lowest roughness values exhibited by the 50 v/v % propolis concentration.

**Conclusions:**

Modification of Amalgomer CR with 50 v/v % propolis may increase its mechanical properties without compromising its esthetic. *Clinical Significance*. Modification of Amalgomer CR by 50 v/v % propolis is supposed to be a hopeful restorative material with favorable characteristics.

## 1. Introduction

Currently, the maximum prevention and minimally invasive approaches in the dental field are the primary concern for the development of new techniques and materials. Accordingly, atraumatic restorative treatment (ART) has been developed depending on the elimination of caries with hand instruments and restoring the tooth with an adhesive substance [1].

Glass ionomer cement (GIC) is the preferred material due to its excellent properties such as low cytotoxicity, potentiality for hard tissues regeneration, low coefficient of thermal expansion, good adhesion to moist tooth structure, and anticariogenic properties because of the fluoride ion release [2, 3]. GIC has also an acceptable biocompatibility and antimicrobial activity [4]. Due to this antimicrobial potentiality, the association of known antimicrobial agents, like propolis, chlorhexidine, and antibiotics to GICs, has been widely studied [5–7]. In specifically difficult clinical conditions as in ART, it may be of a large importance [8].

In spite of these desirable properties, low tensile strength, fracture toughness, and brittleness of the existing conventional types limit their utilization in high-stress situations as in classes I and II restorations [2, 3, 9, 10]. Accordingly, a variety of modifiers have been applied to conventional glass ionomers to improve their mechanical characteristics. Such modifications included the addition of “bioactive” components as glasses and hydroxyapatite or incorporating metal particles or fibers to the composition.[11].

In 1977, incorporation of amalgam alloy powder into glass ionomer was expected to increase the strength and to provide radio-opacity. However, metal-reinforced types of cement have not been used as tooth-colored restoration. Also, the lack of interfacial bonding, which is important for good transport of stress from the matrix in the metal-reinforced GIC, can illustrate why metal-reinforced types of cement have not shown to be of much more strength and durability than their metal-free counterparts [12]. In the late 1980s, the addition of polymerizable hydrophilic resins to conventional glass ionomer cements gave rise to the development of resin-modified formulas. They exhibited better mechanical properties than conventional glass ionomers. Till now, their polymerization shrinkage and low wear resistance are the main drawbacks [13].

Recently, a new ceramic-reinforced glass ionomer (Amalgomer CR) has been brought to the dental market. This tooth-colored product has been advocated by the manufacturer to combine both high strength of a metallic restorative and the advantages of glass ionomers including esthetic [12].

Recently, the use of naturally obtainable products for pharmacological applications has seen a worldwide expansion. Propolis, identified as bee glue, is a natural nontoxic resinous adhesive material. It is obtained by honeybees during mixing the secretions of their hypopharyngeal glands with the digested substance of resins obtained from leaves, flowers of plants, trees, and certain barks, that is utilized as a sealant and sterilizer in honeybee nests. Propolis has been shown to exhibit antioxidant activity, antibacterial, antifungal, antiviral, antitumor, and anti-inflammatory properties [14]. It has a wide variety of uses in dentistry proposed by numerous studies [15–17] such as decrease in dentinal permeability and hypersensitivity, prohibition of carious lesions, decrease in the inflammation of oral mucosa subjected to chemotherapeutic regimens, oral cancer, gingivitis, and periodontitis, a component of dentifrice to manage oral microbiota by its anti-inflammatory effect and direct pulp capping, and as an analgesic material. Moreover, as an antiviral, it retards the growth and development of skin alterations at the beginning of infection with herpes simplex.

Despite these benefits, there are only a few reports regarding the addition of propolis to different dental materials [7]. These researches have concerned about antimicrobial effects, but physical properties have been ignored [18]. Moreover, there is a shortage of reports on the use of ethanolic extract propolis (EEP) form, which possesses numerous pharmacological characteristics.[8] This study aimed to investigate the efficacy of EEP, added to ceramic-reinforced glass ionomer (Amalgomer CR), regarding physicomechanical properties. The null hypothesis was that there is no difference in material behavior concerning color change, surface roughness, hardness, and compressive strength, with or without propolis.

## 2. Materials and Methods

### 2.1. Preparation of Propolis Extract

Pristine propolis used in the present study was acquired from honeybees (*Apismellifera* L) in Mansoura, Egypt. After freezing propolis samples at −20°C, they were ground (ZM 200, Retsch, Haan, Germany) and placed in bottles of 25 g portion. After that, each 25 g of propolis was dissolved in 250 mL of ethanol 80% (vol/vol) at room temperature using a magnetic stirrer for about 24 hours. Then, the extract of propolis was cleared from harsh using a filter to generate EEP. Purified propolis samples were kept in dark at room temperature till being utilized [19].

### 2.2. Preparation of Propolis Containing Amalgomer

The material used in the study is Amalgomer CR (Advanced Health Care, Ltd., Tonbridge, UK, Lot no. 071724-3). Amalgomer liquid was blended with EEP in proportions of 25 and 50 v/v %.

A total of 120 specimens were used to assess color stability, compressive strength, surface microhardness, and surface roughness, 30 specimens for each test. In each test, specimens were equally divided into three groups; (I) Amalgomer CR (control) prepared from the conventional Amalgomer CR liquid, (II) 25 v/v % EEP-modified Amalgomer CR, and (III) 50 v/v % EEP-modified Amalgomer CR (10 specimens each). Moreover, nine representative specimens (three specimens for each test) were used for Fourier-transform infrared spectroscopy (FTIR), X-ray diffraction analysis (XRD), scanning electron microscopy (SEM), and energy-dispersive X-ray analysis (EDX) (one representative specimen for each group).

### 2.3. Specimen Preparation

A sectional Teflon mold (8 mm diameter × 2 mm thickness) was utilized to fabricate disc-shaped specimens used for color stability, surface roughness, and microhardness tests. At the same time, a stainless steel split mold (4 mm in diameter and 6 mm in height) according to ISO standard was utilized to prepare cylindrical specimens for compressive strength testing.

Each mold was first positioned above a glass plate and a Mylar strip. The materials were mixed according to their manufacturers' instructions and placed into the molds. The Mylar strip was placed on each mold, and a second glass plate was then positioned over the mold with a small pressure exerted to extrude the excess material and produce a standardized surface finishing. The excess material that was extruded around the edge of the mold was carefully removed by using a surgical blade. All specimens were stored in deionized water at 37 ± 1°C to equilibrate for 48 hours before testing.

### 2.4. Fourier-Transform Infrared Spectroscopy (FTIR)

Fourier-transform infrared absorption spectra were recorded for 32 runs for three representative specimens for the control and two test groups within the spectral range extended from 4000 to 400 cm^−1^ using single-beam spectrophotometer (Nicolet iS10, Thermo Electron Corporation, UK). The spectral resolution was 2 cm^−1^. Specimens' spectra were corrected for dark current spectra and background. KBr disc technique was used, at which 1 : 100 sample to KBr was used to obtain clear homogenous disks of diameter 1 cm under pressure up to 5 tons/cm [2].

### 2.5. X-Ray Diffraction (XRD)

Three specimens representing the studied groups were used to examine the internal arrangement of atoms within the prepared matrices using XRD diffractogram pattern recorded via the computerized PA analytical X'Pert PRO X-ray machine-adopted Cu K*α* line. The machine was working with 45 kV and 40 mA current. All measurements were performed within Bragg's diffraction angle (2*θ*) ranging between 5 and 70° and wavelength *λ* = 1.540 Å. Peak maxima located at the Bragg angle was utilized to identify the crystalline phases inside the material structure.

### 2.6. Scanning Electron Microscopy (SEM) and Energy-Dispersive X-Ray (EDX)

Morphology and surface nature of three specimens representing the studied groups were examined using a scanning electron microscope (SEM) (JEOL, JSM-6510LV, Japan) linked to the EDX unit operating with accelerating potential 30 kV and magnification up to ×10^6^. All specimens were coated with a thin layer of gold to minimize the effect of charge.

### 2.7. Color Stability

The color of thirty specimens (10 specimens for each group) was assessed using a spectrophotometer (Easyshade®, Vita Zähnfabrik, Bad Säckingen, Germany) employing CIE (Commission International de l'Éclairage) *L*^*∗*^*a*^*∗*^*b*^*∗*^ related to standard illuminant A using a white background as a reference. Standard illuminants provide a foundation for comparing pictures or colors measured under various lighting conditions.[20]. Measurements under natural daylight were conducted. For each sample, measurements were repeated three times, and the average *L*^*∗*^, *a*^*∗*^, and *b*^*∗*^ was evaluated.

The color variation between the color at baseline (control group) and the color of EEP-modified groups (Δ*E*^*∗*^) for each disc specimen was estimated using the following equation [21]:

(1) $$\begin{array}{c} \Delta E^{*}={\left[{\left(\Delta L^{*}\right)}^{2}+{\left(\Delta a^{*}\right)}^{2}+{\left(\Delta b^{*}\right)}^{2}\right]}^{1/2}, \end{array}$$

where values of Δ*E*^*∗*^ ≥ 3.3 were estimated as clinically disagreeable[22].

### 2.8. Compressive Strength

Compressive strengths were determined by using a method similar to that described by ADA [23]. Compressive strength testing for thirty specimens (10 specimens for each group) was carried out using a universal testing machine (Model 3345, Instron Corporation, Canton, MA, USA) at 0.5 mm/min crosshead speed in which load was applied in the long axis of the specimens. The peak stress applied to the fracture of the specimens was recorded, and the compressive strength (CS) (MPa) was calculated as follows:

(2) $$\begin{array}{c} \text{CS}=\frac{4P}{\pi D^{2}}, \end{array}$$

where *P* is the maximum applied load at fracture (N) and *D* is the diameter of the specimen (mm).

### 2.9. Surface Microhardness

Using a Vickers microhardness measuring instrument (HMV Microhardness Tester, Shimadzu, Japan), the surface microhardness of the upper surfaces of thirty specimens (10 specimens for each group) was evaluated. A 200 g load with a dwelling moment of 15 s was implemented through the indenter. For each specimen, five measurements were registered, and the average value of Vickers' hardness was measured and expressed in kg/mm [2].

### 2.10. Surface Roughness

A total number of thirty specimens (ten specimens for each group) were used for the surface roughness testing. Using a surface profilometer (Surftest 211, Mitutoyo, Tokyo, Japan), the surface roughness of each specimen was explored in five distinct locations. The surface roughness cutoff value was 0.8 mm, and the stylus' traversing range was 4 mm. The tracing diamond tip radius was 5 *μ*m, and the measuring strength and velocity were 4 mN (0.4 g) and 0.5 m·s^−1^, respectively. Each sample shows the average roughness value (Ra, *μ*m) as the mean of the Ra values measured in five distinct locations.

Data were subjected to one-way variance analysis (ANOVA) and multicomparison testing by Tukey (*p* < 0.05).

## 3. Results

### 3.1. FTIR Optical Absorption Spectra

**Figure 1:**
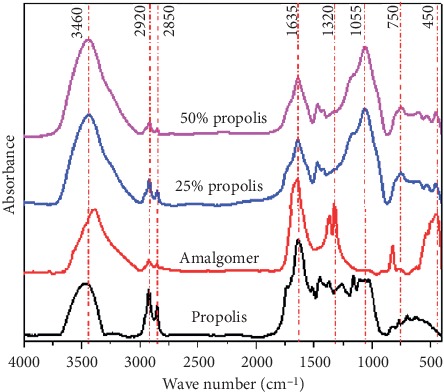
FTIR Absorption spectra of the studied specimens.

Figure 1 reveals FTIR optical absorption spectra of propolis filler, parent Amalgomer CR sample, and their variable volume fraction composite materials.

It was observed that FTIR spectra of propolis filler were characterized by sharp bands belonging to the organic portion of the propolis constituent. The band at about 346 cm^−1^ was assigned for the water and OH vibrational groups, the band at 2920 cm^−1^ was attributed to the asymmetric vibrations, the band at about 1635 cm^−1^ resulted from the overlapping of amide I (C=O) and C=C stretching of methacrylate group, the band at 1320 cm^−1^ might result from aromatic skeletal joined with C–H in plane deforming and stretching, the sharp intense band at 1055 assigned to stretching vibrations of both (C–O–C) or, (C–F), the low intensity band at 750 cm^−1^ was related to the presence of stretching vibrations of both (C–H) and (C–Cl) groups, and finally the bands between 500 and 400 cm^−1^ could be assigned to metal ion contaminations even in ppm level [24].

FTIR spectra of Amalgomer sample and samples that contain different volume fractions of propolis show the identity of glass ionomer cement represented by the main strong band located at about 1055 cm^−1^ attributed to Si–O–Si vibrations from SiO_2_-containing fillers, while the bands in the region 400–600 cm^−1^ can be related to their respective metallic partner. Samples that contain 25 or 50 v/v % propolis/Amalgomer composite show a clear variation, especially in the midregion [25, 26].

### 3.2. X-Ray Diffraction

**Figure 2:**
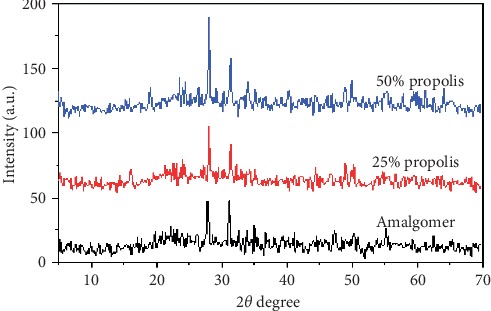
X-ray diffraction pattern of parent Amalgomer CR specimen and variable volume fraction composite materials containing different ratios of propolis.

Figure 2 shows the X-ray diffraction pattern of parent Amalgomer CR sample and variable volume fraction composite materials containing different ratios of propolis, namely, 25 and 50 v/v %.

### 3.3. Scanning Electron Microscopy

**Figure 3:**
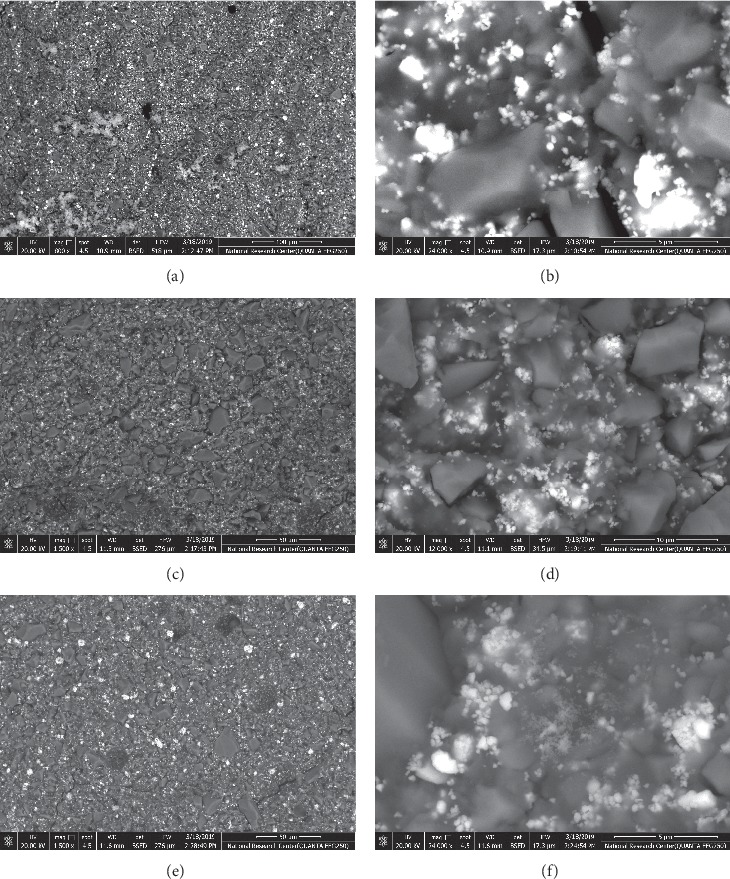
Scanning electron micrographs of studied groups' samples at low (a, c, and e) and high magnifications (b, d, and f).

Figure 3 shows the SEM (×800) micrographs of the surface morphology of representative specimens for the control, 25 and 50 v/v % EEP-modified Amalgomer groups at lower magnification as well and higher magnifications. The existence of some pores and air voids is most obvious in the surface morphology of the control specimen (A, B). The surface morphology of 25 v/v % EEP-modified Amalgomer group exhibited nonuniform distribution of propolis particles between the ceramic and glass particles dispersed in the matrix phase of Amalgomer (C and D). Meanwhile, with a greater increase in the concentration of propolis (50 v/v %), the surface morphology becomes more homogenous and uniform with apparent occlusion of the surface microspores with propolis particles (E, F).

### 3.4. EDX Analysis and Mapping

**Figure 4:**
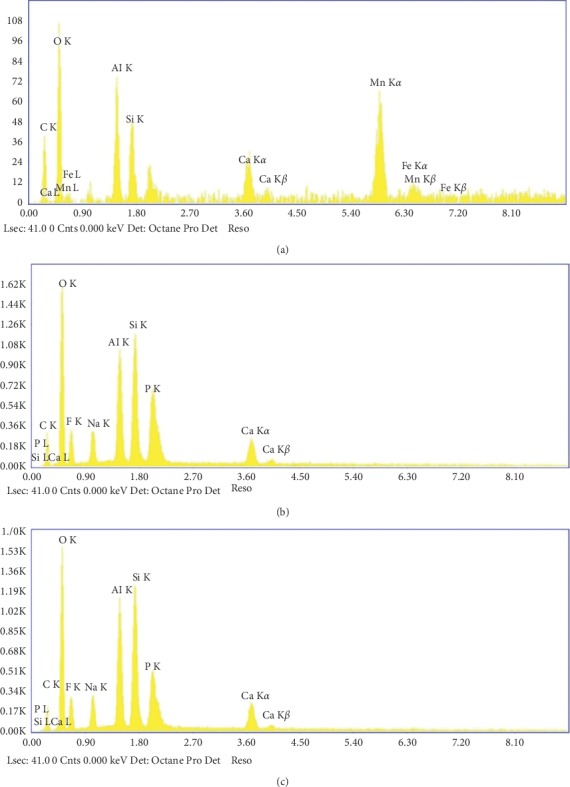
EDX analysis of the main Amalgomer CR specimens before and after addition of different volume proportions of propolis. (a) Amalgomer CR. (b) Amalgomer CR with 25 v/v % propolis. (c) Amalgomer CR with 50 v/v % propolis.

**Figure 5:**
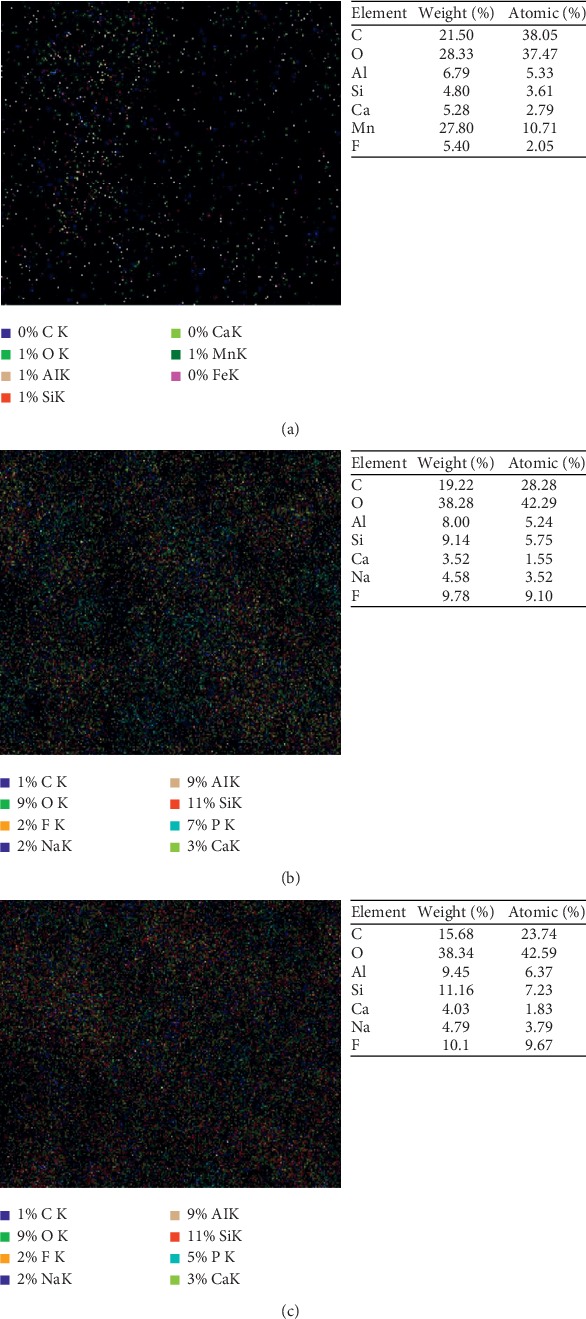
Mapping analysis of the main Amalgomer CR specimens before and after addition of different volume proportions of propolis. (a) Amalgomer CR. (b) Amalgomer CR with 25 v/v % propolis. (c) Amalgomer CR with 50 v/v % propolis.

Figure 4 represents EDX analysis of the main Amalgomer CR specimens before and after addition of different volume fractions of propolis. It was clear that the main inorganic constituent of the parent sample (Si, Al, F, Mn, and Ca) persist in their position except that of Mn ions. The disappearnce of Mn partner after adding propolis may be attributed to the presence of excess fluorine ions in the composition of propolis that may form different shapes of complexes and masking the Mn ions causing a drastic structural change and consequently, in other physical characteristics of studied specimens. Such a behavior was previously recognized by Plenio [27]. This behavior indicates a type of interaction between main constituent and filler. The degree of homogeneity between constituents was approved using mapping images (Figure 5). In addition, it was observed that the total percent of fluoride content tends to increase gradually with increasing propolis content in the sample leading to further changes in the physical characteristics.

### 3.5. Color Stability

Mean color difference values (Δ*E*^*∗*^) and standard deviations of the studied groups are illustrated in Table 1. Group III had insignificantly higher color difference value (1.84 ± 0.17) compared with group II (1.42 ± 0.07), (*p* > 0.05). The color difference values of both groups are clinically acceptable (Δ*E*^*∗*^ ≤ 3.3).

### 3.6. Compressive Strength

Mean compressive strength values and standard deviations for the studied groups are presented in Table 1. Group III demonstrated the highest mean compressive value (86.01 ± 4.34).

While the control group had the lowest value (44.67 ± 3.65), the ANOVA test indicated a significant difference between the two studied EEP groups and the control one (*p* < 0.0001); meanwhile, no significant difference was identified between both EEP-modified Amalgomer CR groups.

### 3.7. Surface Microhardness

Mean surface microhardness values and standard deviations for the considered groups are shown in Table 1. The highest mean microhardness value was exhibited by group III (97.09 ± 4.14), while the control group had the lowest value (64.54 ± 3.21). The ANOVA test indicated a significant difference among the studied groups (*p* < 0.0001), in which 50 v/v % EEP-modified group mean was significantly different from both control group and 25 v/v % EEP-modified Amalgomer CR; however, no significant difference was identified between control and 25 v/v % EEP-modified Amalgomer CR groups.

### 3.8. Surface Roughness

Mean surface roughness values (Ra) and standard deviations for the studied groups are shown in Table 1. The results indicated that group II exhibited the highest mean value (0.92 ± 0.06), while group III had the lowest one (0.57 ± 0.05). The ANOVA test showed a significant difference among the three studied groups (*p* < 0.0001).

## 4. Discussion

Dentistry is concerned about various technologies to evolve new materials and techniques to dental restorative materials [28]. According to this target, a combination of existing materials with various substances gave rise to a promising field. However, further research work has to be performed to evoke this real application. According to this principle, propolis as a simple naturally obtainable product seems to be a good choice in treatment modalities in dentistry. Yet, few studies declared this up till now [7, 29].

Lately, a new ceramic-reinforced glass ionomer (Amalgomer CR) has been launched to the dental market. This tooth-colored product is suggested by the manufacturer to combine the high strength of metallic restorations and the aesthetics and other advantages of glass ionomers [12].

Kouidhi et al. [30] declared that EEP is effective against *S mutans*, *S mitis*, *S oralis*, *S pyogenes*, *S sanguis*, *S salivarius*, *S constellatus*, and *Gemella morbillorum*. Moreover, in a study by Erdem et al. [19], it was found that modifying GIC with EEP increases the antibacterial potential of GIC.

The large ceramic particles reinforcing glass ionomer of Amalgomer CR may participate in its high fluoride discharge [31]. Consequently, propolis incorporation into amalgomer might be beneficial in increasing its antibacterial activity even more than that of its incorporation into GIC. However, no previous studies have reported the effect of its incorporation on physicomechanical properties of Amalgomer.

Regarding physicomechanical properties of Amalgomer CR, the results of this study demonstrated that the null hypothesis could not be accepted, since the modification of Amalgomer CR with EEP altered its physicomechanical properties.

Concerning the color stability test and according to based fundamental of literature, values of Δ*E* < 1 are considered as not perceptible by the human eye. Values of 1 < Δ*E* < 3.3 are regarded as noticeable by skilled operators but clinically satisfying, whereas values of Δ*E* > 3.3 are regarded as detectable by nonskilled persons and are, therefore, clinically disagreeable [32].

The results of this study showed insignificantly higher color difference values of 50 v/v % EEP-modified Amalgomer CR group in comparison to those of 25 v/v % group (*p* > 0.05). This could be attributed to the yellowish discoloration nature of propolis extract which increases with the increase in propolis concentration dissolved in ethanol.

The results of compressive strength test exhibited significantly higher values of both EEP-modified groups (25 and 50 v/v %) when compared with the control group with the highest value corresponding to the higher concentration (50 v/v %). This could be explained by the fact that adding propolis to the liquid of Amalgomer decreases the viscosity of the liquid and extends the working time [31]. This facilitates and enhances the ability of the acid to attack the glass particles. This will improve the rate at which the ions are leached and liberated from the glass. As a consequence, a more fast cement development by gelation of the insoluble products of the acid and basic glass takes place [33]. This explanation is consistent with that of Algera et al. [34] who found that the lower viscosity of the liquid leads to increase of the rate of the reaction by declustering glass particles and improving the dispersion of the reaction components. Hence, mechanical properties could be enhanced [34].

According to the hardness test, the 50 v/v % of EEP-modified group demonstrated significantly higher hardness values in comparison to both 25 v/v % EEP-modified group and the control one. This might be attributed to the large ceramic particles reinforcing glass ionomer of Amalgomer CR which participate in its high microporosities since the particles are not tied to the cement matrix, thus increasing the valuable surface area accessible to be incorporated by propolis particles which may occlude these microporosities especially with higher concentrations (50 v/v %) of EEP. As a consequence, the surface resistance to indentation will increase with a corresponding increase in mechanical properties as well. This explanation supports compressive strength results. The previous clarification is supported by Bahadure et al. [35] who noticed that the large silver alloy particles in a metal-reinforced glass ionomer, that are not linked to the cement matrix, produce an increase in the microporosity of the cement [35].

Roughness results of this study is coincident with the explanation of hardness test results since there was a significant difference between all the tested groups with the lowest mean roughness values demonstrated by the highest concentration of the 50 v/v % EEP-modified group. The higher the concentration of propolis, the higher the ability of its particles in the extract to occlude the microporosities present in the structure of Amalgomer CR matrix as a result of its coarse ceramic particles not being bound to the cement matrix. The scanning electron micrographs of the tested groups support roughness results, where the greatest homogeneity and uniformity in the surface morphology were observed with the greatest concentration of the EEP-modified Amalgomer group.

In contrast, Topcuoglu et al. [36] found that EEP should be present in a small ratio as possible, as the EEP does not participate in the construction of the glass ionomer network of Amalgomer CR, and thus high ratios of EEP would weaken the scaffold and deteriorate the physical properties [36].

## 5. Conclusions

Based on the results presented and within the limitations of this study, the following could be deduced:

1. Amalgomer CR modified with either 25 or 50 v/v % EEP exhibited a nonsignificant color change.
2. Modification of Amalgomer CR with 25 or 50 v/v % EEP improved significantly its surface microhardness and compressive strength.
3. Modification of Amalgomer CR with 50 v/v % EEP significantly decreased its surface roughness.
4. Addition of propolis generates an increase in fluorine content combined with formation of different complexes with the resultant variation of physical characteristics.
5. Amalgomer CR modified with 50 v/v % EEP is promising restorative dental material with enhanced physicomechanical properties. It provides better physicomechanical properties compared with the lower concentration (25 v/v %) of EEP. However, further studies are needed to assess its clinical performance as well as fluoride release and its bond to the tooth structure.

## Figures and Tables

**Table 1:** Means and standard deviations of the physicomechanical properties of Amalgomer CR incorporated with EEP and Tukey's analysis.

Group	Color stability (Δ*E*)	Compressive strength (MPa)	Microhardness (kg/mm^2^)	Surface roughness (*μ*m)
I (control)	—	44.67 ± 3.65^b^	64.54 ± 3.21^b^	0.67 ± 0.07^b^
II (25 wt% EEP)	1.42 ± 0.07	84.22 ± 60^a^	68.28 ± 1.80^b^	0.92 ± 0.06^a^
III (50 wt% EEP)	1.84 ± 0.17	86.01 ± 4.34^a^	97.09 ± 4.14^a^	0.57 ± 0.05^c^
*p* value	0.1	<0.0001	<0.0001	<0.0001

Mean values with same superscripted letters are significantly different at *p* < 0.05.

## Data Availability

All data presented or analyzed during this study are included within this article.

## References

[1] Wang L., Lopes L. G., Bresciani E., Lauris J. R. P., Mondelli R. F. L., Navarro M. F. L. (2004). Evaluation of Class I ART restorations in Brazilian schoolchildren: three-year results. *Special Care in Dentistry*.

[2] Shiekh R. A., Rahman I. A., Masudi S. M., Luddin N. (2014). Modification of glass-ionomer cement by incorporating hydroxyapatite silica nanopowder composite: sol-gel synthesis and characterization. *Ceramics international*.

[3] Li S. Q., Su B. H., Ran J. G., Wang J., Yan L. L., Tu X. M. (2015). Effects of niobium oxide addition on the mechanical properties of glass ionomer cement. *Materials Science Forum*.

[4] Cefaly D. F. G., Wang L., Mello L. L. C. P. d., Santos J. L. d., Santos J. R. d., Lauris J. R. P. (2006). Water sorption of resin-modified glass-ionomer cements photoactivated with LED. *Brazilian Oral Research*.

[5] Ferreira H. C., Rego M. A. (2006). Evaluation in vitro of properties physicist-chemistries of glass-ionomer cements, after addition of propolis and antibiotics. *CiêncOdontolBras*.

[6] Sanders B. J., Gregory R. L., Moore K., Avery D. R. (2002). Antibacterial and physical properties of resin modified glass-ionomers combined with chlorhexidine. *Journal of Oral Rehabilitation*.

[7] Yesilyurt C., Er K., Tasdemir T., Buruk K., Celik D. (2009). Antibacterial activity and physical properties of glass-ionomer cements containing antibiotics. *Operative Dentistry*.

[8] Troca V. B. P. B., Fernandes K. B. P., Terrile A. E., Marcucci M. C., Andrade F. B. d., Wang L. (2011). Effect of green propolis addition to physical mechanical properties of glass ionomer cements. *Journal of Applied Oral Science*.

[9] Zoergiebel J., Ilie N. (2013). Evaluation of a conventional glass ionomer cement with new zinc formulation: effect of coating, aging and storage agents. *Clinical Oral Investigations*.

[10] Zraikat H. A., Palamara J. E., Messer H. H., Burrow M. F., Reynolds E. C. (2011). The incorporation of casein phosphopeptide-amorphous calcium phosphate into a glass ionomer cement. *Dental Materials*.

[11] Wang Y., Darvell B. W. (2009). Hertzian load-bearing capacity of a ceramic-reinforced glass ionomer cement stored wet and dry. *Dental Materials*.

[12] Neveen M. A., Salwa A. E., Osama M. B. (2008). An in-vitro study of the physico-mechanical properties of a new esthetic restorative versus dental amalgam. *Revista de Clínica e Pesquisa Odontológica*.

[13] Deepa G., Shobha T. (2010). A clinical evaluation of two glass ionomer cements in primary molars using atraumatic restorative treatment technique in India: 1 year follow up. *International Journal of Paediatric Dentistry*.

[14] Russo A., Cardile V., Sanchez F., Troncoso N., Vanella A., Garbarino J. A. (2004). Chilean propolis: antioxidant activity and antiproliferative action in human tumor cell lines. *Life Sciences*.

[15] Mahmoud A. S., Almas K., Dahlan A. A. (1999). The effect of propolis on dentinal hypersensitivity and level of satisfaction among patients from a university hospital Riyadh, Saudi Arabia. *Indian Journal of Dental Research*.

[16] Ghisalbert E. L. (1979). Propolis: a review. *Bee World*.

[17] Scheller S., Ilewicz L., Luciak M., Skrobidurska D., Stojko A., Matuga W (1978). Biological properties and clinical application of propolis. IX. Experimental observation on the influence of ethanol extract of propolis (EEP) on dental pulp regeneration. *Arzneimittel-Forschung*.

[18] Rezende G. P. S. R., Pimenta F. C., Costa L. R. R. S. (2006). Antimicrobial activity of two brazilian commercial propolis extracts. *Brazilian Journal of Oral Sciences*.

[19] Erdem H., Fırat O., Tugca B., Sertac A., Neslihan S. (2014). Antibacterial and mechanical properties of propolis added to glass ionomer cement. *The Angle Orthodontist*.

[20] CIE Standard. Standard Method of Assessing the Spectral Quality of Daylight Simulators for Visual Appraisal and Measurement of Colour, A standard method for assessing the quality of daylight simulators, ISO Standard 23603:2005(E)

[21] Sirin Karaarslan E., Bulbul M., Yildiz E., Secilmis A., Sari F., Usumez A. (2013). Effects of different polishing methods on color stability of resin composites after accelerated aging. *Dental Materials Journal*.

[22] Ruyter I. E., Nilner K., Möller B. (1987). Color stability of dental composite resin materials for crown and bridge veneers. *Dental Materials*.

[23] Cho G. C., Kaneko L. M., Donovan T. E., White S. N. (1999). Diametral and compressive strength of dental core materials. *The Journal of Prosthetic Dentistry*.

[24] Hussein U., Hassan N., Elhalwagy M. (2017). Ginger and propolis exert neuroprotective effects against monosodium glutamate-induced neurotoxicity in rats. *Molecules*.

[25] Khan A. S., Khalid H., Sarfraz Z. (2017). Vibrational spectroscopy of selective dental restorative materials. *Applied Spectroscopy Reviews*.

[26] Yamakami S. A., UBALDINI A. L. M., Sato F., Medina Neto A., Pascotto R. C., Baesso M. L. (2018). Study of the chemical interaction between a high-viscosity glass ionomer cement and dentin. *Journal of Applied Oral Science*.

[27] Plenio H. (2004). The coordination chemistry of fluorine in fluorocarbons. *Chembiochem*.

[28] Bertolini M. J., Zaghete M. A., Gimenes R., Padovani G. C. (2008). Determination of the properties of an experimental glass polyalkenoate cement prepared from niobium silicate powder containing fluoride. *Dental Materials*.

[29] Özan F., Sümer Z., Polat Z. A., Er K., Özan Ü., Değer O. (2007). Effect of mouthrinse containing propolis on oral microorganisms and human gingival fibroblasts. *European Journal of Dentistry*.

[30] Kouidhi B., Zmantar T., Bakhrouf A. (2010). Anti-cariogenic and antibiofilms activity of Tunisian propolis extract and its potential protective effect against cancer cells proliferation. *Anaerobe*.

[31] Bhattacharya A., Vaidya S., Anil K., Tomer A., Raina A. (2017). GIC at its best-a review on ceramic reinforced. *GIC*.

[32] Yap A. U., Sim C. P., Loh W. L., Teo J. H. (1999). Human-eye versus computerized color matching. *Operative Dentistry*.

[33] Woolford M. J. (1994). Effect of radiant heat on the surface hardness of glass polyalkenoate (ionomer) cement. *Journal of Dentistry*.

[34] Algera T. J., Kleverlaan C. J., de Gee A. J., Prahl-Andersen B., Feilzer A. J. (2005). The influence of accelerating the setting rate by ultrasound or heat on the bond strength of glass ionomers used as orthodontic bracket cements. *European Journal of Orthodontics*.

[35] Bahadure R., Pandey R., Kumar R., Gopal K., Singh R. (2012). An estimation of fluoride release from various dental restorative materials at different pH: in vitro study. *Journal of Indian Society of Pedodontics and Preventive Dentistry*.

[36] Topcuoglu N., Ozan F., Ozyurt M., Kulekci G. (2012). *In vitro* antibacterial effects of glass-ionomer cement containing ethanolic extract of propolis on Streptococcus mutans. *European Journal of Dentistry*.

